# Supplementary material for: Audiologist’s Perspective in Auditory Rehabilitation: Implications for Ethical Conduct and Decision-Making in Portugal
Source: Audiol Res. 2022 Mar 26;12(2):171–81. doi: 10.3390/audiolres12020020 (PMC9028603; doi:10.3390/audiolres12020020)
Supplement: Supplementary file 1 [file audiolres-12-00020-s001.zip › Questionnaire A (English version).pdf]

Audiologist´s ethical conduct and decision-making survey

The present study is part of a Research Project of the Institute of Bioethics in collaboration with the Otorhinolaryngology Clinic of the Faculty of Medicine of the University of Coimbra. The purpose of the study is to explore the decision-making process and ethical concerns in auditory rehabilitation in Portugal. We ask you to respond genuinely and truthfully. For this purpose, we request the collaboration of an Audiologist with professional experience of 1 year or more in the field of auditory rehabilitation.
Over time, some doubts may be felt in answering. However, it would be important to answer all questions, always giving the answer that best represents you, making sure you don't leave it blank. The maximum duration for execution is 20 minutes.
The questionnaire is individual and must be answered autonomously.
In case of doubt, you can contact the principal investigator by email tatiana.marques@estescoimbra.pt.
Your contribution is valuable not only for Audiology as a profession but also for the practice of auditory rehabilitation in Portugal.
Thank you for your cooperation!

* Mandatory answer

This questionnaire is anonymous.
The survey question record does not contain any information about your identity, unless any survey question asks for identification and you provide it.
If you used a code to access this survey, this code will not be saved with your answers. The code is managed in a separate database and is only used by the program to record that it has completed the survey. There is no way to relate the codes of those invited to participate in the survey to the answers given.

Informed Consent

* I agree to participate in the Audiologist´s ethical conduct and decision-making survey , as part of a Research Project of the Biomedical Institute of the Faculty of Medicine of the University of Coimbra in collaboration with the Otorhinolaryngology Clinic of the Faculty of Medicine of the University of Coimbra.

The nature and objectives of the study were explained to me and informed about the possibility of clarifying all aspects that seem relevant to me. I was also guaranteed the possibility of withdrawing from participating in the study, whenever I wanted to.

My identity will never be revealed and the data provided will remain confidential. I agree that they are analyzed by the principal investigator for the study and its collaborators, under the authority of the principal investigator.

I agree to participate and authorize the data from the completed online questionnaire to be used for research purposes.

Choose the option if you agree to participate in the study *

○ I have understood the information provided to me and I intend to proceed to participate in the study.

Part 1: Personal Information

What is your gender? *

Choose one of the following options

- ◯ Female
- ◯ Male

What is your age? *

A sua resposta

What is your marital status? *

Choose one of the following options

- ◯ Married
- ◯ Cohabitation
- ◯ Not married

What is your higher degree of education *

Choose one of the following options

- ◯ Undergraduate
- ◯ Master's degree
- ◯ Doctorate

What is your region of practise? *

Choose one of the following options

- ◯ North
- ◯ Center
- ◯ Lisbon and Tejo Valley
- ◯ Alentejo
- ◯ Algarve
- ◯ Archipelago of Madeira
- ◯ Archipelago of Azores

For how long has you worked as Audiologist in the field of auditory rehabilitation? *

Choose one of the following options

- ◯ 1 to 4 years
- ◯ 5 to 9 years
- ◯ more than 10 years

Part 2: Ethical concerns and decision-making

Carefully read the following questions and select the one that best describes your opinion.

Which factor do you consider decisive to advise the patient in the auditory rehabilitation process? *

Choose one of the following options

- ◯ Audiometric tests
- ◯ Clinical experience
- ◯ Patient opinion
- ◯ Colleague's opinion
- ◯ Professional guidelines
- ◯ Manufacturers guidelines

In a scenario where the optimal solution is not clear or there is conflicting information or contraindication for the patient's auditory rehabilitation, which situation do you look for to make the decision? *

Choose one of the following options

- ◯ Client's goals and preferences
- ◯ Discussion with the colleague's
- ◯ Discussion with the supervisor
- ◯ Trial and error
- ◯ Previous experience
- ◯ Manufacturer or employer incentives

What professional guidelines do you usually use? *

- ☐ APtA
- ☐ ASHA
- ☐ BSA
- ☐ Other
- ☐ None

How often do you use the guidelines of professional associations (APtA, ASHA, BSA or others)? *

A sua resposta

How difficult is it when you have to make a decision about the process indicated to rehabilitate the patient. *

Rate the following option according to how important it is to you

	1	2	3	4	5	
Not at all difficult	◯	◯	◯	◯	◯	Very difficult

Rate the following option on the confidence you have in your decisions in your clinical practice. *

Choose one of the following options

	1	2	3	4	5	
Not at all confident	○	○	○	○	○	Complete confident

In your experience, which of the following alternatives has caused you ethical concerns in clinical practise? *

- ☐ Academic aspects
- ☐ Compliance with regulatory guidelines
- ☐ Interactions with colleagues or with the supervisor
- ☐ Clinical aspects
- ☐ Interactions between family and caregiver
- ☐ Financial incentives
- ☐ Other
- ☐ N/A

Submit your testimonial statement about an ethical dilemma or decision-making difficulties you faced.

A sua resposta

In your clinical practice, do you receive incentives from the manufacturers or from your employer? *

Choose one of the following options

- ○ Yes
- ○ No

What kind of incentives do you receive? *

Choose one of the following options

- ◯ Fixed value per hearing aid fitted
- ◯ According to the number of hearing aids fitted
- ◯ Varied with the manufacturer of hearing aids
- ◯ Varied the technology of hearing aids
- ◯ Others
- ◯ N/A

Select the option(s) that you have experienced in your clinical practice. *

- ☐ Gave incomplete or incorrect information to the patient
- ☐ Endangered the patient
- ☐ Committed tax fraud
- ☐ Other
- ☐ N/A

Rate the following option on the influence of the supervisor on your decision-making. *

Choose one of the following options

	1	2	3	4	5	
Not at all influent	◯	◯	◯	◯	◯	Extremely influent

Rate the following option on the influence of the supervisor on your decision-making. *

Choose one of the following options

	1	2	3	4	5	
Not at all influent	◯	◯	◯	◯	◯	Extremely influent

Does your supervisor or employer encourage working with unqualified professionals? *

Choose one of the following options

- Yes
- No

If you answered yes to the previous question, please describe the ethical dilemmas you faced in your work related to unqualified colleagues?

A sua resposta

Thank you for your participation!
