# Supplementary material for: Audiologist’s Perspective in Auditory Rehabilitation: Implications for Ethical Conduct and Decision-Making in Portugal
Source: Audiol Res. 2022 Mar 26;12(2):171–81. doi: 10.3390/audiolres12020020 (PMC9028603; doi:10.3390/audiolres12020020)
Supplement: Supplementary file 1 [file audiolres-12-00020-s001.zip › Questionnaire B (Portuguese version).pdf]

Questões éticas na reabilitação auditiva

O presente estudo insere-se no âmbito de um Projeto de Investigação do Instituto de Bioética em colaboração com a Clínica de Otorrinolaringologia da Faculdade de Medicina da Universidade de Coimbra.
O objetivo do estudo consiste em explorar os desafios éticos enfrentados pelos Audiologistas na área da Reabilitação Auditiva em Portugal. Para que o estudo seja o mais fiel à realidade atual, pedimos-lhe que responda com genuidade e veracidade. Para o efeito, solicitamos a colaboração de Audiologistas com experiência profissional em reabilitação auditiva superior a 1 ano, para o preenchimento de um questionário online.
Ao longo do preenchimento do questionário poderá sentir dificuldade em responder a algumas questões. Contudo, seria importante que respondesse a todas as questões, dando sempre a resposta que lhe pareça mais adequada. Agradecemos que tente responder a todas as questões, assegurando-se que não deixa nenhuma questão em branco. A duração máxima prevista para concluir o questionário é de 20 minutos.
O questionário é individual, devendo ser respondido de forma autónoma.
O questionário é totalmente anónimo e confidencial, não sendo requerida qualquer informação que permita identificar os participantes. A participação no estudo é voluntária, sendo-lhe garantida a possibilidade de desistência em qualquer momento do preenchimento do questionário, se assim o entender.
Em caso de dúvidas, pode contactar o investigador principal pelo email tatiana.marques@estescoimbra.pt.
O seu contributo é extremamente valioso não só para a Audiologia como profissão como para a prática da reabilitação auditiva em Portugal.
Muito obrigado pela sua colaboração!

*Obrigatório

Este questionário é anónimo.
O registo das perguntas ao inquérito não contém qualquer informação sobre a sua identidade, excepto se alguma pergunta do inquérito solicitar alguma identificação e a fornecer.
Se usou um código para aceder a este inquérito este código não será guardado junto com as suas respostas. O código é gerido numa base de dados separada e apenas é utilizado pelo programa para registar que concluiu o inquérito. Não há forma de relacionar os códigos dos convidados a participar no inquérito com as respostas dadas.

Consentimento informado

* Concordo em participar no estudo "Questões éticas na reabilitação auditiva", integrado no âmbito de um Projeto de Investigação do Instituto de Biomédica da Faculdade de Medicina da Universidade de Coimbra em colaboração com a Clínica de Otorrinolaringologia da Faculdade de Medicina da Universidade de Coimbra.

Foram-me explicados a natureza e objetivos principais do estudo e informada sobre a possibilidade de esclarecer todos os aspetos que me pareçam pertinentes. Foi-me também garantida a possibilidade de desistir de participar no estudo, sempre que assim o entenda.

A minha identidade nunca será revelada e os dados fornecidos se manterão confidenciais. Concordo que os mesmos sejam analisados pela investigadoras investigadora principal pelo estudo e colaboradores do mesmo, sob autoridade da investigadora principal.

Concordo em participar e autorizo que os dados do questionário online preenchido sejam utilizados para fins de investigação.

Escolha a opção se aceita participar no estudo. *

- [ ] Compreendi a informação que me foi fornecida e pretendo prosseguir para participar no estudo.

Parte 1: Informação Pessoal

Sexo *

Escolha uma das seguintes opções

- [ ] Feminino
- [ ] Masculino

Idade *

Escolha uma das seguintes opções

A sua resposta

Estado Civil *

Escolha uma das seguintes opções

- [ ] Solteiro(a)
- [ ] Casado(a)
- [ ] Em união de facto/coabitação
- [ ] Divorciado
- [ ] Viúvo

Habilitações Literárias *

Escolha uma das seguintes opções

- [ ] Bacharelato
- [ ] Licenciatura
- [ ] Mestrado
- [ ] Doutoramento

Área de residência *

Escolha uma das seguintes opções

- ◯ Norte
- ◯ Centro
- ◯ Lisboa e Vale do Tejo
- ◯ Alentejo
- ◯ Algarve
- ◯ Arquipélago da Madeira
- ◯ Arquipélago dos Açores

Há quanto tempo exerce funções como Audiologista na área de Reabilitação Auditiva? *

Escolha uma das seguintes opções

- ◯ Menos de 1 ano
- ◯ Entre 1 e 4 anos
- ◯ Entre 5 e 9 anos
- ◯ Mais de 10 anos

Parte 2: Questões éticas e tomada de decisão

Leia atentamente as seguintes afirmações e selecione a que descreve melhor a sua opinião.

Qual o factor que considera decisivo para aconselhar o paciente no processo de reabilitação auditiva? *

Escolha uma das seguintes opções

- ◯ Testes audiométricos
- ◯ Experiência do Audiologista
- ◯ Opinião do Cliente
- ◯ Directrizes da Associação Portuguesa de Audiologistas
- ◯ Opinião dos Colegas
- ◯ Directrizes dos Fabricantes

Numa situação em que a solução optimizada para o paciente não é clara, qual das situações procura para tomar a decisão? *

Escolha uma das seguintes opções

- ◯ Preferências e objectivos do paciente
- ◯ Discussão do caso com colegas
- ◯ Discussão do caso com supervisor
- ◯ Tentativa e erro
- ◯ Experiências anteriores
- ◯ Incentivos da empresa ou do fabricante

Quais as directrizes para o exercício da reabilitação auditiva que recorre habitualmente? *

- ☐ APtA
- ☐ ASHA
- ☐ BSA
- ☐ Outra
- ☐ Nenhuma

Quantas vezes recorre às directrizes das associações profissionais (APtA, ASHA, BSA)? *

A sua resposta

Qual o grau de dificuldade quanto tem que tomar uma decisão quanto ao processo indicado para reabilitar o paciente. *

Classifique a seguinte opção quanto à importância que apresenta para si

	1	2	3	4	5	
Nada difícil	◯	◯	◯	◯	◯	Extremamente difícil

Classifique a seguinte opção quanto à confiança que apresenta nas suas decisões na sua prática clínica. *

	1	2	3	4	5	
Nada confiante	○	○	○	○	○	Completamente confiante

Na sua experiência profissional, qual(is) da(s) seguinte(s) categoria(s) considera que é susceptível de despoletar dilemas éticos. *

- [ ] Académicos
- [ ] Conformidade com as directrizes de associações profissionais
- [ ] Colegas de trabalho/supervisores
- [ ] Critérios clínicos
- [ ] Família/cuidador
- [ ] Incentivos financeiros
- [ ] Outra(s)
- [ ] Não aplicável

Dê o seu testemunho de um dilema ético ou dificuldade na tomada de decisão que tenha vivenciado na sua prática clínica.

A sua resposta

Na sua prática clínica recebe incentivos financeiros? *

- ○ Sim
- ○ Não

Na sua prática clínica que tipo de incentivos recebe? *

- ○ valor fixo por reabilitação auditiva
- ○ quantidade de aparelhos auditivos adaptados
- ○ variável com o fornecedor dos aparelhos auditivos adaptados
- ○ variável com a tecnologia dos aparelhos auditivos adaptados
- ○ Outro(s)
- ○ Não aplicável

Seleccione a(s) opção(ões) que vivenciou na sua prática clínica. *

- ☐ disponibilizou informação incompleta ou imprecisa ao paciente
- ☐ colocou em perigo o paciente
- ☐ cometeu fraude fiscal
- ☐ Outra
- ☐ Não se aplica

Qual a influência do seu supervisor no seu aconselhamento e reabilitação dos pacientes? *

	1	2	3	4	5	
Nada influente	○	○	○	○	○	Extremamente influente

O seu supervisor ou empresa, incentiva o trabalho com audioprotesistas ou profissionais não qualificados? *

- ○ Sim
- ○ Não

Se respondeu sim à pergunta anterior, comente os dilemas éticos inerentes à sua prática clínica em colaboração com estes profissionais?

A sua resposta

Obrigado pela sua participação!
